# Low postoperative complication rate with high survival rate and good clinical outcome 9 years after autologous chondrocyte transplantation of the knee joint

**DOI:** 10.1007/s00402-022-04611-1

**Published:** 2022-10-05

**Authors:** Yannick J. Ehmann, Thekla Esser, Amr Seyam, Marco-Christopher Rupp, Julian Mehl, Sebastian Siebenlist, Andreas B. Imhoff, Philipp Minzlaff

**Affiliations:** 1 Department of Orthopedic Sports Medicine, Technical University of Munich, Ismaninger Str. 22, 81675 Munich, Germany; 2 Department of Orthopedic Sports Medicine, Orthoclinic Agatharied, Agatharied, Germany

**Keywords:** ACT, ACI, Autologous chondrocyte transplantation, Autologous chondrocyte implantation, Long term outcome, Early revision, Early complication, Survival rate, Survival

## Abstract

**Purpose:**

To investigate postoperative complications and associated risk factors for failure following autologous chondrocyte transplantation (“ACT”) as well as its long-term survival and clinical function. It was hypothesized that ACT is a safe technique for cartilage repair with a low incidence of postoperative complications and rare rates of revision surgery combined with a high long-term survival and good to excellent clinical outcome in long-term-follow-up.

**Methods:**

All patients undergoing ACT-Cs of the knee joint between 2006 and 2012 at the author’s institution were included in this retrospective study. Concomitant procedures had been performed if necessary. Early postoperative complications, revision surgeries, failure and risk factors for those events were evaluated 6 months after the surgery. Long-term clinical outcome was assessed using the Lysholm Score, the Tegner Score, a 10-grade scale for satisfaction and the Visual Analogue Scale (VAS) at a minimum follow-up of 9 years postoperatively. Long-term survival was calculated using revision surgeries, clinical failures and conversion procedures to create a Kaplan–Meier analysis. A subgroup analysis for different defect locations was performed. 139 patients were included in this study (27% female/ 73%male; age 26.7 [21.7; 35.2] years). The median defect size was 4.0 [3.0; 6.0] cm^2^ (40% medial femoral condyle (MFC), 17% lateral femoral condyle (LFC), 36% patella, 19% trochlea). 97 (70%) of the patients had undergone previous surgery and 84 (60%) underwent concomitant procedures.

**Results:**

Postoperatively, 8% of patients had complications (4% bleeding, 2% arthrofibrosis, 2% infection), 7% of patients needed revision surgery. 12% of patients had a prolonged deficit in ROM, that did not require revision surgery. No significant difference in terms of complications was found between the patellofemoral and femorotibial group. Patients demonstrated good patient reported long-term outcomes 9–15 years after the index surgery (Tegner: 4.7 ± 1.8; VAS: 2.4 ± 2.1; Lysholm: 80 ± 14; satisfaction with operation: 7.3 ± 1.9). Survival rates were 88% at 9 years, 85% at 11 years, and 85% at 13 years after the index procedure. Reasons for failure included debridement of ACT (*n* = 4; 5%), revision ACT (*n* = 3, 3%), conversion to total knee arthroplasty (*n* = 3, 3%) and conversion to High tibial osteotomy (HTO) (*n* = 1; 1%)).

**Conclusion:**

The present study indicates ACT as an effective treatment option for femorotibial- as well as patellofemoral cartilage defects with a high long-term survival and low conversion rate as well as good long-term results regarding knee function and satisfaction. Postoperative complications needing revision surgery are rare. Prolongated deficits of range of motion appear frequently up to six months especially in patellofemoral defects, but can often be successfully addressed by intensified physiotherapy without requiring an arthrolysis.

**Level of evidence:**

Level III.

## Introduction

Due to low self-healing potential, full-thickness cartilage defects in the knee are a risk factor for further joint deterioration, the development of osteoarthritis and the need for a future total knee arthroplasty (TKA). The risk is increased with increased defect size [9]. Cartilage repair procedures can not only reduce the progression of these focal defects, but also of the total knee joint degeneration [13]. Autologous cartilage transplantation (ACT) is an important and well-established regenerative treatment option for middle to large (> 2.5 cm^2^) full-thickness chondral and osteochondral defects in the knee [28] which can successfully rebuild hyaline or hyaline-like cartilage [7, 19, 28, 29].

Short- and medium-term outcome of first generation ACT were promising [7], as well as the long-term outcome regarding pain reduction und function improvement [25, 28, 29]. Nevertheless, graft hypertrophy, disturbed fusion, delamination and insufficient regeneration present complications of the early generation ACT [10, 21]. These complications were reduced with technical improvements in higher generations of ACT (ACT-C, MACI) [4, 5, 21] showing predominantly good to excellent clinical outcome [8, 16].

While current ACT technique shows satisfying short- and middle term clinical results especially for patients with isolated cartilage defects [22, 26], there is a scarcity of data describing immediate postoperative complications and the long-term outcome, which are of clinical relevance.

The purpose of this study was to investigate postoperative complications and associated risk factors for failure following ACT-Cs in different defect locations in the knee joint as well as long-term survival and clinical function. We hypothesised, that ACT-Cs is a secure technique for cartilage repair with a low incidence of postoperative complications and rare rates of revision surgery combined with a high long-term survival and good to excellent clinical outcome in long-term follow-ups.

## Materials and methods

Ethical approval was obtained via Human Research Determination Form to the institutional review board (IRB) of the Technical University of Munich (IRB #DKZ5476). All patients undergoing surgery for ACT-Cs between 2006 and 2012 at the author’s institution were included in this retrospective study. Surgeries were performed by or under supervision of a highly specialized knee surgeon. Patients with femorotibial defects received an a.p. full-leg radiography to detect malalignments. In case of a varus- or valgus malalignment of more than 2° a concomitant valgus or varus producing osteotomy was performed to unload the affected compartment. In case of knee or patella instabilities, ligaments reconstruction was performed.

### Clinical outcome

Follow-up was performed at 2 points. An early-stage-follow-up was performed 6 months postoperatively, a long-term follow-up was performed 9 to 15 years after the initial surgery.

Complications that occurred up to the early-stage follow-up were defined as ‘postoperative complications’. Complications included arthrofibrosis with the need for arthroscopic arthrolysis, postoperative hemarthrosis with the need for lavage or joint aspiration and infection (soft tissue- and knee joint infection) within the first 6 months. Early revision surgery as a consequence of any postoperative complications were reported. Patients with “prolongated deficit of Range of Motion (ROM)” (defined as extension < 0°/flexion < 90°) after 6 months combined without the need for arthrolysis during the short-term and long-term follow-up were reported, separately, but not included as postoperative complications, if no revision surgery was necessary.

A second stage-follow up was performed in 2021, 9–15 years after initial surgery, to evaluate the long-term survival and clinical outcome. Patients were assessed regarding any further revision surgeries or conversion procedures (e.g., the implantation of a TKA) and the long-term survival of the ACT-Cs was recorded. A Kaplan–Meier curve was used to estimate the probability of failure and survival as a function of time. Long-term clinical outcome was evaluated using the Lysholm Score, the Tegner Score and the Visual Analogue Scale (VAS). [32] Postoperative satisfaction was evaluated with a 10-grade scale (‘‘very satisfied = 10 points, ‘‘dissatisfied’’ = 0 points) according to the VAS. The patients were contacted via questionnaire and telephone. For all patients who could not be contacted in the second-stage follow-up, a chart review was performed to gain information about postoperative complications, failures, conversion procedures and revision surgeries.

### Surgical technique

Surgery was performed in a two-step approach, as described before by Brittberg [7]. In the first surgery, patients underwent an arthroscopy to determine localization, size and degree of the chondral defect. Surrounding cartilage was assessed. Healthy cartilage specimens were harvested and sent to a laboratory. The chondrocytes were then isolated and proliferated in vitro for 3–5 weeks.

In a second surgical procedure, open ACT-Cs was performed using a medial or lateral arthrotomy under tourniquet control. After debridement of the defect, the defect size was measured. The Chondro-Gide^®^ Membrane was cut into size. After resuspending, chondrocytes were applied to the rough side of the membrane. A waiting time of 10 min was admitted for adhesion, before the membrane was placed into the defect, the chondrocyte-seeded side facing the defect**.** The membrane was fixed with 5.0 PDS sutures and Fibrin glue in the suture areas. If necessary, concomitant procedures like Anterior Cruciate Ligament (ACL)/Medial Patello Femoral Ligament (MPFL) reconstruction, High Tibial Osteotomy (HTO) or meniscus surgery were performed during the same operation.

### Postoperative rehabilitation

Each patient underwent a structured rehabilitation protocol for a minimum of 3 months postoperatively. Patients were under strict immobilization for 48 h postoperatively. Rehabilitation began on the third postoperative day under the direction of a trained physical therapist and using a Continuous Passive Motion (CPM) machine. Patients were asked to wear a brace with limited range of motion (ROM) for 6 weeks. ROM was limited to extension/flexion 0/0/30° for 2 weeks, 0/0/60° for weeks 3–4, 0/0/90° for weeks 5–6 in patellofemoral defects and to extension/flexion 0/0/90° for 6 weeks in femorotibial defects. Weight bearing was limited to 20 kg for 6 weeks. Cryotherapy and lymphatic drainage were applied to reduce pain and edema. Full pivoting sporting activities were not allowed for 6 months, contact sporting activities were not allowed for 9 months.

### Statistical analysis

Statistical Analysis was performed using IBM SPSS Statistics for Windows, version 27.0 (IBM Corp., Armonk, NY, USA). For categorical data, the non-parametric Pearson’s chi-square (*χ*^2^) test or, if sample size was too small, the Fisher’s exact test was used to investigate an association between potential risk factors (gender, smoking behavior, previous surgery, defect localization) and the outcome measures postoperative complications and early revision surgery. If appropriate, the correlation between the named variables and postoperative complications/early revision surgery was determined using the contingency coefficient (*C*). Metric parameters (defect size, age) were tested for normal distribution using the Kolmogorov–Smirnov-test. Due to missing normal distribution, the non-parametric Mann–Whitney *U* test was applied to investigate on association with the outcome parameters. A significant result for all applied tests was considered at *p* < 0.05.

## Results

### Patient baseline characteristics

The study cohort comprised of 139 patients (27% female, 73% male). The median patients’ age was 26.7 [21.7; 35.2] years. The median defect size was 4.0 [3.0; 6.0] cm^2^. 97 (70%) of the patients had undergone previous surgery and 84 (60%) underwent concomitant procedures (Tables 1, 2). Concerning the defect localization, 56 patients (40%) had a defect on the medial femoral condyle (MFC), 24 (17%) on the lateral femoral condyle (LFC), 50 (36%) on the patella and 26 (19%) in the trochlea. A subgroup-analysis was performed regarding different defect locations. Patients’ baseline characteristics are listed in Tables 1, 2.

**Table 1:** Patients characteristics of the patient collective

	Overall, *n* = 139 (100%)	Femorotibial, *n* = 64 (46.0%)	Patellofemoral, *n* = 55 (39.6%)	Multiple lesions, *n* = 20 (14.4%)
Gender [*n*(%)]				
Male	102 (73%)	49 (77%)	37 (67%)	16 (80%)
Female	37 (27%)	15 (23%)	18 (33%)	4 (20%)
Age [years]	26.67 [21.67; 35.17]	28.29 [21.13; 35.29]	24.50 [21.42; 31.67]	34.08 [24.67; 41.25]
Smokers [*n*(%)]	49 (35%)	22 (34%)	22 (40%)	5 (25%)
Defect localization [*n*(%)]				
MFC	56 (40%)*	45 (70%)		11 (55%)*
LFC	24 (17%)*	19 (30%)		5 (25%)*
Patella	50 (36%)*		41 (75%)	9 (45%)*
Trochlea	26 (18%)*		14 (25%)	12 (60%)*
Defect size [cm^2^]	4.00 [3.00; 6.00]	4.63 [3.00; 6.00]	4.00 [2.30; 6.00]	6.00 [4.50; 8.00]
s.c. drain [n(%)]	47 (34%)	23 (36%)	20 (36%)	5 (25%)
i.a. drain [n(%)]	47 (34%)	19 (30%)	21 (38%)	7 (35%)

*s.c.* subcutaneous, *i.a.* intraarticular, *MFC* medial femoral condyle, *LFC* lateral femoral condyle

*Defects could be in multiple locations of the knee joint, percentages add up to > 100%

**Table 2:** Previous surgeries and concomitant procedures

	Overall, *n* = 139 (100%)	Femorotibial, *n* = 64 (46%)	Patellofemoral, *n* = 55 (40%)	Multiple lesions, *n* = 20 (14%)
previous surgery [*n*(%)]	97 (70%)	33 (24%)	64 (46%)	47 (73%)
Cartilage repair	34 (62%)			
Others	16 (80%)			
Concomitant procedure [*n*(%)]	84 (60%)	51 (80%)	24 (44%)	9 (16%)
HTO	23 (17%)	17 (27%)	1 (2%)	5 (25%)
ACL Reconstruction	10 (7%)	10 (16%)		
Patella surgery(MPFL suturing/reconstruction, tuberositas osteotomy)	13 (9%)	3 (5%)	9 (45%)	1 (5%)
Others	38 (27%)	21 (33%)	14 (25%)	3 (15%)

*HTO* high tibial osteotomy, *ACL* Anterior cruciate ligament

All patients’ data were included in the early-stage-follow-up (*n* = 139). A retrospective chart-review was performed for all patients and all patients were contacted for long-term-follow-up. 86 patients could be reached and were included in the long-term survival analysis and the long-term clinical outcome analysis.

### Early complications and revision

The study population had a postoperative complication rate of 8% (11 patients): 2% (3 patients) developed an arthrofibrosis, 4% (5 patients) suffered from postoperative bleeding and 2% (3 patients) had a postoperative soft tissue infection, without infection of the knee joint. Early revision surgery rate up until the 6 month follow-up was 7% (10 patients). Arthrolysis was necessary in 2% of patients (3 patients), punction or lavage in 4% (5 patients). Only one patient (1%) received a revision ACT-Cs four months after the primary surgery, due to sclerosis of the primary ACT-Cs and did not report problems 6 months after the revision. One patient (1%) had a subsequent High-Tibial-Osteotomy (HTO) 6 months after the surgery due to pain. In the long term follow up, the patient reported 86 points in the Lysholm score, a Tegner score of 4 and did not need further surgery afterwards (Table 3).

**Table 3:** Patients characteristics of the patient collective. Prolonged deficit of Range of Motion (ROM) is defined as less than Extension/Flexion 0–0–90° after 6 months without the need for arthrolysis during the further follow-up

	Overall, *n* = 139 (100%)	Femorotibial, *n* = 64 (46%)	Patellofemoral, *n* = 55 (40%)	Multiple lesions, *n* = 20 (14%)
Postoperative complication [*n* (%)]	11 (8%)	3 (5%)	5 (9%)	3 (15%)
Arthrofibrosis	3 (2%)	1 (2%)	2 (4%)	
Bleeding infection	5 (4%)		3 (6%)	2 (10%)
Soft tissue infection	3 (2%)	2 (3%)	0 (0%)	1 (5%)
Knee joint infection	–	–	–	–
Early revision surgery [*n* (%)]	10 (7%)	3 (5%)	5 (9%)	2 (10%)
Arthrolysis	3 (2%)	1 (2%)	2 (4%)	–
Punction or lavage	5 (3%)	–	1 (2%)	1 (5%)
Re-ACT-Cs	1 (1%)	1 (2%)	–	–
HTO	1 (1%)	1 (2%)	–	–
Prolonged deficit of range of motion	16 (12%)	3 (5%)	13 (24%)	–

*HTO* high tibial osteotomy

16 patients (11.5%) had a prolongated deficit of Range of Motion (ROM) after 6 months. Of those, 14 of (9%) did not need revision surgery, neither in the first six month, nor in the long-term follow-up. One patient was treated with a HTO 3 years after the primary surgery due to pain, but at that time did not have any limitations in the ROM as described above. One patient was converted to a unicompartimental arthroplasty of the knee 13 years after the primary surgery, due to pain, the ROM normalized after 6 months.

### Subgroup analysis: defect location

**Fig. 1:**
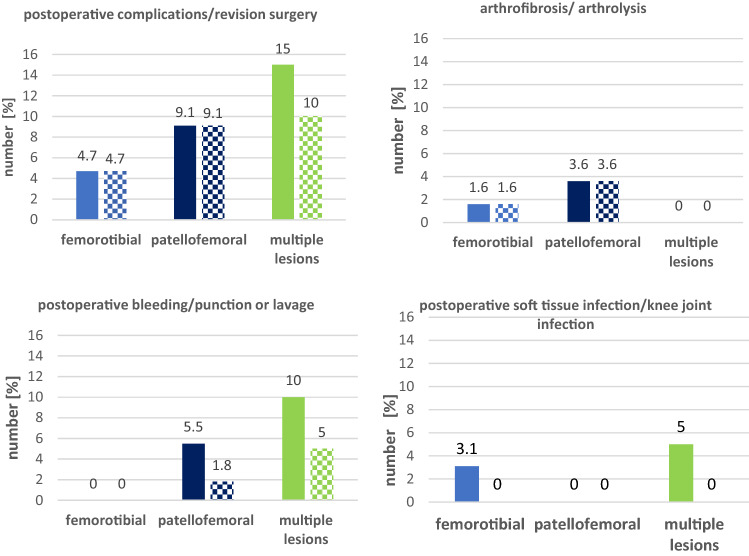
Number of postoperative complication (plain color) and early revision surgery (squared color) in the different locations. *p* values for statistical differences are indicated if *p* < .05

Development of postoperative complications was compared between the different defect localizations. 3 (5%) patients with patellofemoral defects, 5 (9%) with femorotibial defects and 3 (15%) with multiple lesions developed a postoperative complication. No significant difference was found between the different defect localizations (Fig. 1).

**Fig. 2:**
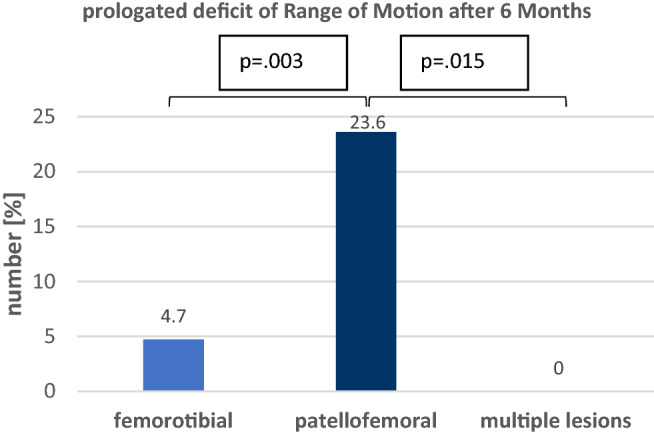
Number of prolongated deficit of Range of Motion (ROM) after 6 months in the different locations. Prolonged deficit of ROM is defined as less than Extension/Flexion 0–0–90 after 6 months without the need for arthrolysis during the further follow-up. P-values for statistical differences are indicated if *p* < 0.05

A prolongated deficit in range of motion was detected in 13 (23.6%) of the patients with a patellofemoral defect and in 3 (4.7%) of the patients with femorotibial defects, while none of the patients with multiple lesions was affected. A significant difference between femorotibial- and patellofemoral defects (*p* = 0.003) with a correlation of *r* = 0.267 (*p* = 0.003) was revealed, furthermore there was a significant difference between patellofemoral defects and multiple lesions (*p* = 0.015) with a correlation of *r* = 0.266 (*p* = 0.017) (Fig. 2).

For early revision surgery, there was no significant difference between the three groups. A significant correlation (*r* = 0.269, *p* = 0.010) was found between femorotibial defects and multiple lesions for postoperative punction or lavage, though showing no significant difference (*p* = 0.055). Results for early revision surgery are shown in Fig. 2.

### Risk factor analysis

**Fig. 3:**
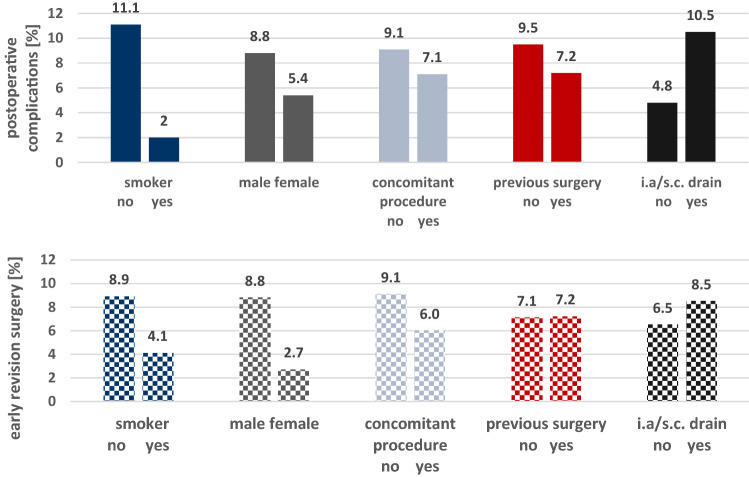
Risk factor analysis for postoperative complications (plain colour) and early revision surgery (squared colour). *p* values for statistical differences are indicated if *p* < 0.05

**Fig. 4:**
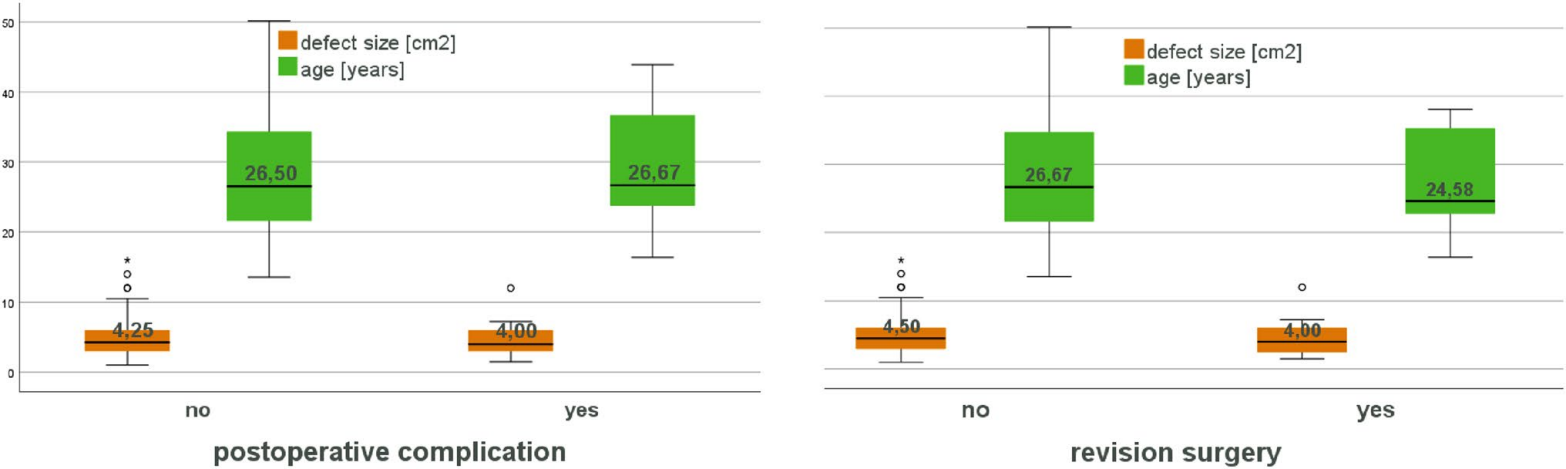
Analysis of postoperative complications and early revision surgery correlation with defect size and age. Values are indicated as median. *p* values for statistical differences are indicated if *p* < 0.05

Potential risk factors for developing postoperative complications or the need to undergo early revision surgery were tested. None of the parameters (patient age, defect size, smoking behaviour, sex, concomitant procedures, previous surgeries or the use of s.c./i.a. drain) were able to predict the development of postoperative complications or the need to undergo an early revision surgery (Figs. 3, 4).

### Long term follow-up

**Fig. 5:**
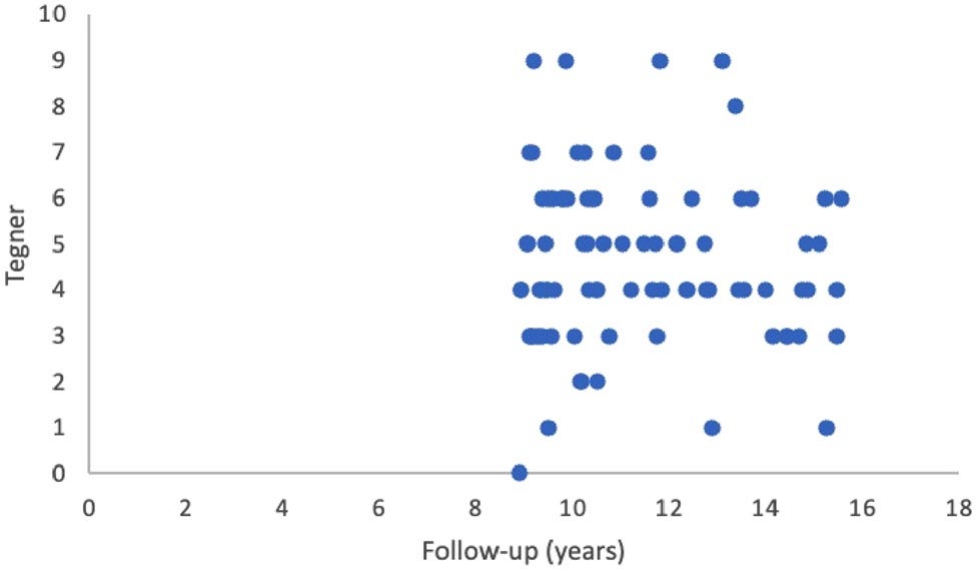
Scatterplot showing the results in Tegner Score over time

**Fig. 6:**
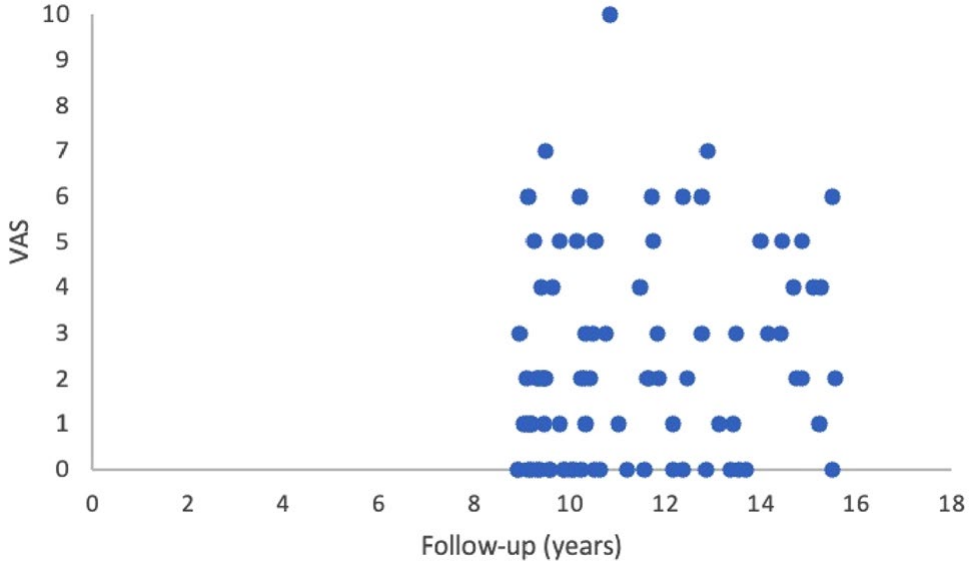
Scatterplot showing the results in VAS over time

86 Patients participated in the long-term-follow-up. There was no significant difference in patients baseline data between the patients who participated in the long-term-follow-up collective compared to the early-stage-follow-up collective regarding age (*p* = 0.421), defect size (*p* = 0.987), number of previous operations (*p* = 0.615), gender (*p* = 0.833), side (*p* = 0.411) or smoking habits (*p* = 0.221). Median time until long-term-follow-up was 11.3 ± 2.1 years, mean age was 40 ± 10 years. Patients were able to achieve favorable results regarding the return to sports and the function of the knee in a long-term follow-up, as shown in Table 4. Figures 5 and 6 show scatterplots of the Lysholm Score, the Tegner score, the VAS and the satisfaction at follow-up.

**Table 4:** Results of the second-stage-follow-up after 9–15 years

	Overall, *n* = 86 (100%)
Patient characteristics	
Follow-up in years	11.3 ± 2.1
Gender (male/female)	62 (72%)/24 (28%)
Age at Follow-up	40 ± 10
Patient results	
VAS	2.4 ± 2.1
Tegner Score	4.7 ± 1.8
Lysholm Score	80 ± 14
Revision Surgery/Failure	11 (13%)
Debridement	4 (5%)
Re-ACTc	3 (3%)
Prothesis	3 (3%)
HTO	1 (1%)
Satisfaction with surgery	7.3 ± 1.9

*VAS* Visual Analogue Scale

**Fig. 7:**
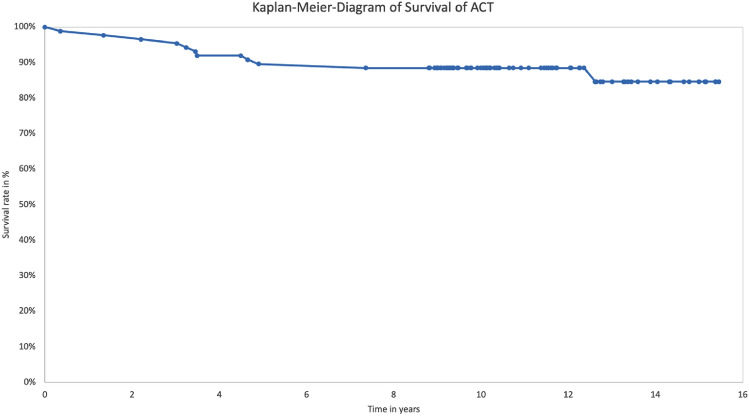
Kaplan–Meier-Diagram showing the long time survival rate of the ACT. Latest follow up of patients is shown with points

The results of the Kaplan–Meier-Analysis of the long-term survival of the ACT-Cs is shown in Fig. 7. Survival rates in this collective were 88% at 9 years, 85% at 11 years, and 85% at 13 years after the index procedure. Reasons for failure are shown in Table 4.

## Discussion

The most important findings of this study are that ACT-Cs is an effective treatment option for femorotibial- as well as patellofemoral cartilage defects with a high long-term survival and low conversion rate. The long-term results regarding knee function and satisfaction are favorable. Postoperative complications needing revision surgery are rare.

Our findings show an overall postoperative complication rate for ACT-Cs of 8%, 7% needed an intervention. Given the complexity of the two-step surgical procedure including open arthrotomy and concomitant procedures this overall complication- and revision-rate can be considered as low, in particular regarding arthrofibrosis, knee joint infection and secondary re-alignement. They can be considered as comparable to recent literature [27]. Current literature reported arthrofibrosis as a major complication after third generation ACT (Novocart 3D), mainly occurring in retropatellar defects [23]. The reported rate of 22% cases of arthrofibrosis with arthrolysis was much higher than ours, where only 2% of the overall study population developed an arthrofibrosis that needed arthrolysis. Furthermore, unspecific postoperative complications have been reported to be rare after ACT-P [28], ACT-C [10] and in third generation-ACT (Novocart 3D) [2] with the rate of arthrofibrosis, bleeding and soft tissue infection being equal to our findings. The present study shows that ACT-Cs has a low complication rate. The rates seem low for an arthrotomy based technique [10] which indicates ACT-Cs to be a safe procedure.

Niemeyer et al. reported four possible major complications after all generations of ACT: symptomatic hypertrophy, disturbed fusion, graft failure and delamination, causing the need of revision surgery in 27% in ACT-P and 12% in ACT-C and 15% in third generation ACT. There is evidence that hypertrophy has decreased with further development of the technique [21]. Until the six-month-follow-up, no failures or cases of hypertrophy and delamination were seen in our study population and no revision for the mentioned reasons above was necessary. However, the mean period of time to revision surgery because of the named symptoms varies between 16 and 20 months in the literature [10, 21]. In the present study only symptomatic patients were assessed concerning postoperative complications and early revision surgery. Furthermore, indication for concomitant HTO was provided strictly leading to a low rate of HTOs after the ACT. Bode et al. found evidence, that an additional HTO in patients with varus deformities < 5° leads to lower rates of revision surgery and longer survival of ACT. [6] They assumed, that the fact of reduced pressure peaks in the medial compartment leads to reduced pressure in the ACT transplant [1]. This, and additionally the relatively short time until the first follow up, might be reasons for the absence of cases of hypertrophy and delamination.

The subgroup analysis regarding the defect location showed that patients with patellofemoral defects suffered from a prolongated deficit in range of Motion more often than patients with femorotibial defects. We believe that the stricter limitation of the ROM in the rehabilitation process of patellofemoral lesions is causal for this finding [11, 23]. We could not identify a significant difference between the different locations for the development of arthrofibrosis—this might be due to the low overall number of arthrofibrosis in this collective. We conclude that prolongated deficits of Range of Motion may appear frequently, especially in patellofemoral defects, but can often be successfully addressed by intensified physiotherapy without needing a surgery.

We could not identify any risk factors to predict postoperative complications or early revision surgery. Current literature supports our findings that patient age and defect size do not have an influence on the incidence of revision surgery [21]. Kon et al. on the other hand, found good results in patients older than 40 years receiving second generation ACT, but nevertheless inferior to the results in younger patients [15]. We could not identify an effect of previous surgeries on the rate of postoperative complication and early revision surgery. The rate of treatment failure in secondary ACT after failed cartilage surgery affecting the subchondral bone (e.g. Microfracture, MFX) seems to be higher than in patients receiving initial ACT [17, 30]. In the present study cartilage repair presented a minor part of previous surgery. This is of clinical relevance as, it might suggest that the cautious use of e.g., MFX procedures is one of the reasons for the low revision rate and good outcome in the present study. This should be taken into account when deciding for the initial type of cartilage repair procedure. Even though we could not identify a higher postoperative complication- or early revision rate in smokers, smoking is known to have an effect on wound healing [18, 31]. We consider the low rate of impaired wound healing (soft tissue infection) in the present study as reason for the absence of a significant difference between smokers and non-smokers. Additionally, it has been proven before that smoking affects the outcome of ACT negatively in terms of function and failure [12]. In this paper we did not correlate smoking behavior with functional outcome and long-term survival, which would have been interesting to compare with the available evidence.

Recent systematic reviews show that ACT can lead to very good results in medium term follow up, but there is a scarcity of data regarding the long term follow up. This study shows that the clinical outcome and the patients satisfaction is high in most patients at a follow up of > 10 years [20]. Ogura et al. showed in their recent study, that ACT in adolescents showed a survival rate of 90% in a 10 year follow up. This study is able to support this findings and show that long term survival in adult patients is high as well [3, 24]. Most studies in the literature that investigate the long-term follow up of ACT only look into small patient populations [3]. This study helps to find more evidence in a larger patient population.

A common limitation of retrospective studies that investigate long-term outcome is that patients are lost to follow up. In this study the average lost to follow up was 3.3% of the patient collective per year (4,6 patients per year). In comparable studies a yearly lost-to-follow-up of up to 5% was reported [34]. The patients’ baseline data of the long-term-follow-up collective showed no significant differences to the early-stage-follow-up-collective. Nevertheless, it cannot be ruled out, that the loss-to-follow-up might have had an effect on the results.

The findings of this study are of high clinical relevance as they might help to realistically evaluate the long-term outcome when discussing chondral transplantation in the day-top-day clinical practice. Especially in young patients the long-term survival is important to evaluate and openly discuss the risk for revision surgery and long-term outcome. It can be discussed with patients that even if there is a prolonged deficit in ROM, an arthrolysis is rarely necessary. The results show that the strict concomitant correction of varus/valgus malalignment, did not lead to a high number of revisions of the osteotomies. This study might help to outline possible outcomes in the patient interaction.

The present study has some limitations. First, not all initially included participants were able to complete the long-term follow-up. A common limitation of retrospective studies that investigate on long-term outcome is that patients are lost to follow up. In this study the average lost to follow up was 3.3% of the patient collective per year (4,6 patients per year). In comparable studies a yearly lost-to-follow-up of up to 5% was reported [34]. The patients’ baseline data of the long-term-follow-up collective showed no significant differences to the early-stage-follow-up-collective. Nevertheless, it cannot be ruled out, that the loss-to-follow-up might have had an effect on the results. Second, the study design is retrospective and no preoperative scores are available. However, all patients were preoperatively highly symptomatic in terms of pain and reduced quality of life with the willingness to undergo a complex two step surgical procedure. After up to 15 years, patients show good clinical scores and are highly satisfied with their outcome. Third, patient reported outcome measurements are excellent tools to evaluate the knee function. VAS, Tegner and Lysholm Score are well accepted and widely used in the orthopedic community. They give the majority of knee surgeons an extensive idea of current knee function. The Lysholm score was originally designed for ligamentous instabilities but is validated and often used in terms of chondral defect treatment [14, 33]. However, radiologic assessments are missing due to ethical concerns and a rating of osteoarthritis cannot be performed.

## Conclusion

The present study indicates ACT-Cs as an effective treatment option for femorotibial- as well as patellofemoral cartilage defects with a high long-term survival and low conversion rate as well as good long-term results regarding knee function and satisfaction. Postoperative complications requiring revision surgery are rare. Prolongated deficits of range of motion appear frequently up to six months especially in patellofemoral defects, but can often be successfully addressed by intensified physiotherapy without requiring an arthrolysis.

## References

[1] Agneskirchner JD, Hurschler C, Wrann CD (2007). The effects of valgus medial opening wedge high tibial osteotomy on articular cartilage pressure of the knee: a biomechanical study. Arthroscopy.

[2] Angele P, Fritz J, Albrecht D (2015). Defect type, localization and marker gene expression determines early adverse events of matrix-associated autologous chondrocyte implantation. Injury.

[3] Barié A, Kruck P, Sorbi R (2020). Prospective Long-term Follow-up of Autologous Chondrocyte Implantation With Periosteum Versus Matrix-Associated Autologous Chondrocyte Implantation: A Randomized Clinical Trial. Am J Sports Med.

[4] Bartlett W, Skinner JA, Gooding CR (2005). Autologous chondrocyte implantation versus matrix-induced autologous chondrocyte implantation for osteochondral defects of the knee: a prospective, randomised study. J Bone Jt Surg Br.

[5] Bentley G, Bhamra JS, Gikas PD (2013). Repair of osteochondral defects in joints–how to achieve success. Injury.

[6] Bode G, Schmal H, Pestka JM (2013). A non-randomized controlled clinical trial on autologous chondrocyte implantation (ACI) in cartilage defects of the medial femoral condyle with or without high tibial osteotomy in patients with varus deformity of less than 5°. Arch Orthop Trauma Surg.

[7] Brittberg M, Lindahl A, Nilsson A (1994). Treatment of deep cartilage defects in the knee with autologous chondrocyte transplantation. N Engl J Med.

[8] Brittberg M, Recker D, Ilgenfritz J (2018). Matrix-Applied Characterized Autologous Cultured Chondrocytes Versus Microfracture: Five-Year Follow-up of a Prospective Randomized Trial. Am J Sports Med.

[9] Everhart JS, Abouljoud MM, Kirven JC (2019). Full-thickness cartilage defects are important independent predictive factors for progression to total knee arthroplasty in older adults with minimal to moderate osteoarthritis: data from the osteoarthritis initiative. J Bone Jt Surg American.

[10] Harris JD, Siston RA, Brophy RH (2011). Failures, re-operations, and complications after autologous chondrocyte implantation—a systematic review. Osteoarthritis Cartilage.

[11] Hirschmüller A, Baur H, Braun S (2011). Rehabilitation after autologous chondrocyte implantation for isolated cartilage defects of the knee. Am J Sports Med.

[12] Jaiswal PK, Macmull S, Bentley G (2009). Does smoking influence outcome after autologous chondrocyte implantation?: a case-controlled study. J Bone Jt Surg Br.

[13] Jungmann PM, Gersing AS, Baumann F (2019). Cartilage repair surgery prevents progression of knee degeneration. Knee Surg Sports Traumatol Arthrosc.

[14] Kocher MS, Steadman JR, Briggs KK (2004). Reliability, validity, and responsiveness of the Lysholm knee scale for various chondral disorders of the knee. J Bone Jt Surg Am.

[15] Kon E, Filardo G, Condello V (2011). Second-generation autologous chondrocyte implantation: results in patients older than 40 years. Am J Sports Med.

[16] Kreuz PC, Kalkreuth RH, Niemeyer P (2019). Long-term clinical and MRI results of matrix-assisted autologous chondrocyte implantation for articular cartilage defects of the knee. Cartilage.

[17] Lamplot JD, Schafer KA, Matava MJ (2018). Treatment of failed articular cartilage reconstructive procedures of the knee: a systematic review. Orthop J Sports Med.

[18] Manassa EH, Hertl CH, Olbrisch R-R (2003). Wound healing problems in smokers and nonsmokers after 132 abdominoplasties. Plast Reconstr Surg.

[19] McCarthy HS, Roberts S (2013). A histological comparison of the repair tissue formed when using either Chondrogide(®) or periosteum during autologous chondrocyte implantation. Osteoarthritis Cartilage.

[20] Mistry H, Connock M, Pink J (2017). Autologous chondrocyte implantation in the knee: systematic review and economic evaluation. Health Technol Assess.

[21] Niemeyer P, Pestka JM, Kreuz PC (2008). Characteristic complications after autologous chondrocyte implantation for cartilage defects of the knee joint. Am J Sports Med.

[22] Niemeyer P, Lenz P, Kreuz PC (2010). Chondrocyte-seeded type I/III collagen membrane for autologous chondrocyte transplantation: prospective 2-year results in patients with cartilage defects of the knee joint. Arthroscopy.

[23] Niethammer TR, Niethammer T, Valentin S (2015). Revision surgery after third generation autologous chondrocyte implantation in the knee. Int Orthop.

[24] Ogura T, Bryant T, Minas T (2017). Long-term outcomes of autologous chondrocyte implantation in adolescent patients. Am J Sports Med.

[25] Ossendorff R, Franke K, Erdle B (2019). Clinical and radiographical ten years long-term outcome of microfracture vs. autologous chondrocyte implantation: a matched-pair analysis. Int Orthop.

[26] Pestka JM, Bode G, Salzmann G (2014). Clinical outcomes after cell-seeded autologous chondrocyte implantation of the knee. Am J Sports Med.

[27] Pestka JM, Luu NH, Südkamp NP (2018). Revision surgery after cartilage repair: data from the German cartilage registry (KnorpelRegister DGOU). Orthop J Sports Med.

[28] Peterson L, Minas T, Brittberg M (2000). Two- to 9-year outcome after autologous chondrocyte transplantation of the knee. Clin Orthop Relat Res.

[29] Peterson L, Brittberg M, Kiviranta I (2002). Autologous chondrocyte transplantation. Biomechanics and long-term durability. Am J Sports Med.

[30] Schuette HB, Kraeutler MJ, Schrock JB (2020). Primary autologous chondrocyte implantation of the knee versus autologous chondrocyte implantation after failed marrow stimulation: a systematic review. Am J Sports Med.

[31] Sørensen LT, Hemmingsen U, Kallehave F (2005). Risk factors for tissue and wound complications in gastrointestinal surgery. Ann Surg.

[32] Tegner Y, Lysholm J (1985). Rating systems in the evaluation of knee ligament injuries. Clin Orthop Relat Res.

[33] Vavken P, Samartzis D (2010). Effectiveness of autologous chondrocyte implantation in cartilage repair of the knee: a systematic review of controlled trials. Osteoarthritis Cartilage.

[34] Wignadasan W, Chang JS, Kayani B (2021). Long-term results of revision total knee arthroplasty using a rotating hinge implant. Knee.

